# Safeguarding the Milk Supply

**Published:** 1922-05

**Authors:** Wilfred Buckley

**Affiliations:** C.B.E., Chairman of the National Clean Milk Society


### SAFEGUARDING THE MILK SUPPLY.

By **WILFRED BUCKLEY, C.B.E., Chairman of the National Clean Milk Society.**

ON August 4, 1914, the day that war was declared in Europe, the Milk & Dairies Act, 1914, was passed by the House of Commons. Within a few days it was accepted by the House of Lords and received the signature of His Majesty. In 1915 a further Bill was passed, the Milk & Dairies (Consolidation) Act, 1915, which made no alterations in the existing laws, but, as its title denotes consolidated in one Act the Act of 1914 with those sections of previous Acts which had not been repealed. Owing to the war, it was found necessary to postpone putting into operation the Act of 1915 until not later than one year from the termination of the War. The War ended legally at the end of August 1921, hence unless further legislative action be taken the 1915 Act must be put in force not later than the end of August of this year.

During the War period further experience was gained in regard to the milk situation. The Government had to pay closer attention than it had in the past to all questions relating to our food supply, and in this respect milk was not neglected.

As a result of the war-time experience those Departments chiefly concerned, the Ministries of Food, of Health and of Agriculture, agreed upon the needed reforms, and in consequence the Milk Amendment Bill was introduced in the House of Commons on May 10, 1920, by the Minister of Health and seconded by the present Minister of Agriculture, who was then Parliamentary Secretary to that Ministry. The main provisions of that Bill were :—

(i) Provision for the licensing of dairymen and dairies (clause 1).

(ii) An extension of the provisions of the Principal Act in regard to the classification of milk (clause 2).

(iii) Provision for the undertaking by sanitary authorities, with the approval of the Minister of Health, of the supply and distribution of milk, or the cleansing, storage, etc., of milk for sale by dealers (clause 6).

(iv) The constitution of Milk and Dairies Committees by the local authorities who will administer the Act (clause 7).

Clause 6, which gave local authorities power under certain conditions to deal in milk, met, naturally, with the strenuous opposition of the trade, and the consequence was that a measure which the Government had framed and introduced in the interests of public health was heard of no further, not, be it noted, because of any change of view by our guardians of public health, but because it was in some respects unacceptable to interests upon which the public have to rely for their milk supply. There are about forty thousand retailers of milk in the country and forty million consumers !

During the last two years a change in the situation has been taking place so far as producers and distributors are concerned. They are beginning to realise, through the wise leadership of some of their members whose mental horizon is not bounded by the past, that it is to their own interest that more liquid milk should be consumed by the nation. They know that there is room for expansion in this respect because they are told that the *per capita* consumption of liquid milk in the United States is three times what it is in England, and they notice that the British consumption of condensed and dried milks, which are chiefly imported articles, increases, while that of liquid milk does not. They are beginning to appreciate that the *only* way to increase consumption is by offering the public a better article, something that is more palatable and wholesome—a liquid food that the people will like to drink.

The public, too, is beginning to appreciate that milk is an extremely valuable food, that in hygienic quality our supply is not good and that it could and should be better. The third party concerned, those responsible for our public health, as represented by the Ministry of Health, are aware of the growing desire on the part of the consumers as well as that of producers and distributors that something should be done, and in consequence there is reason to anticipate that the Government will now take the legislative action that is needed to permit or encourage the needed improvements for which the situation is ripe.

The Act of 1915 must be postponed, revoked, amended or put into operation on or before August 31 of this year. That is the situation as it stands at the present moment. That Act is based on coercive methods ; it relies chiefly upon inspection. There are, perhaps, more than 200,000 farmers producing milk and 40,000 firms distributing it. How thorough an inspection of premises and of methods, made sufficiently frequently, could be organised ? How many inspectors would be required ? Where are a sufficient number of men trained in dairy hygiene to be found ? Where could the nation find the money to pay for them ? Could such a system be put into force, we should still fail to have clean, wholesome milk. I. believe that the improvement that we all desire can only be effected by a system that combines education with encouragement.

In my opinion, a Milk Amendment Bill should be introduced which will provide for a system of classification of milk so that consumers may know just what kind of milk they are getting, and producers and distributors may be paid for the article they sell in accordance with its merits. If milk be classified on the basis of its hygienic quality at any time before reaching the consumer, in our cities and towns a single inspector could obtain a large number of samples in less time and at less expense than would be entailed in visiting and inspecting a single farm. A further necessity is that powers should be given (as in the Milk and Dairies (Scotland) Act, 1915, so that certificates of registration granted to all milk sellers may be revoked or suspended if such a course is in the interests of public health, any holder of a certificate feeling aggrieved having power of appeal to a higher authority.

The Government must now decide what it intends to do. It must put into operation, postpone, revoke or amend the Act of 1915. I doubt if a Minister of Health would either wish or dare to revoke or even to postpone that Act. I think that those concerned with legislation are convinced that the Act is unworkable in its present form and can be improved upon, and that it should not be put into operation in its present form, but should be amended.

Whether or not the Government takes action, I presume will depend to some degree upon the attitude of Members of Parliament, and their attitude in turn depends upon that of their constituents. We may be sure that the unprogressive milk seller will impress upon his Member that he does not want to be shifted from his old rut. The question remains as to how many of the public who want a more wholesome milk supply, in their own interests and in those of other people, will take the trouble to acquaint their Members with their wishes.

After all, we get in this world just about what we deserve, and we shall continue to get a poor milk supply so long as each one of us does not do what he or she can to improve it.

